# Supplementary material for: The American Friendship Project: A report on the status and health of friendship in America
Source: PLoS One. 2024 Jul 30;19(7):e0305834. doi: 10.1371/journal.pone.0305834 (PMC11288408; doi:10.1371/journal.pone.0305834)
Supplement: S1 File — (DOCX) [file pone.0305834.s001.docx]

| **Variable** | **2022**  **SCRI Sample** | | **2023**  **SCRI Sample** | |
| --- | --- | --- | --- | --- |
| **Income** | **N** | **%** | **N** | **%** |
| Less than $10k a year | 248 | 10.2 | 165 | 7.4 |
| $10-19k a year | 249 | 10.3 | 172 | 7.7 |
| $20-29k a year | 339 | 14.0 | 278 | 12.4 |
| $30-39k a year | 272 | 11.2 | 254 | 11.3 |
| $40-49k a year | 214 | 8.8 | 243 | 10.8 |
| $50-59k a year | 198 | 8.2 | 237 | 10.6 |
| $60-69k a year | 127 | 5.2 | 109 | 4.9 |
| $70-79k a year | 157 | 6.5 | 153 | 6.8 |
| $80-89k a year | 76 | 3.1 | 79 | 3.5 |
| $90-99k a year | 88 | 3.6 | 117 | 5.2 |
| $100-149k a year | 260 | 10.7 | 275 | 12.3 |
| More than $150k a year | 192 | 7.9 | 161 | 7.2 |
| **Education Level** |  |  |  |  |
| Less than High School | 93 | 3.8 | 60 | 2.7 |
| High School or GED | 1019 | 42.1 | 889 | 39.6 |
| Associate Degree (in progress) | 96 | 4.0 | 103 | 4.6 |
| Associate Degree | 319 | 13.2 | 288 | 12.8 |
| Bachelor’s Degree (in progress) | 120 | 5.0 | 121 | 5.4 |
| Bachelor’s Degree | 536 | 22.1 | 527 | 23.5 |
| Advanced Degree (in progress) | 45 | 1.9 | 42 | 1.9 |
| Advanced Degree | 192 | 7.9 | 213 | 9.5 |
|  | | | | |

**Supplemental Table A. AFP 2022 and 2023 Study Demographics Part IV**

**Notes.** 2022 SCRI *N* = 2420. 2023 SCRI *N* = 2243. These questions were not asked of the student sample.

**Supplemental Table B. AFP 2022 Student Study Demographics Part V**

| **Variable** | **Student Sample** | |
| --- | --- | --- |
|  | **N** | **%** |
| **International Student** |  |  |
| Yes | 38 | 3.3 |
| No | 1113 | 96.7 |
| **Years at University** |  |  |
| One Year or Less | 554 | 48.1 |
| Two Years | 289 | 25.1 |
| Three Years | 299 | 26.0 |
| Four Years | 0 | 0 |
| Five+ Years | 10 | .9 |
| **Student Status (*if first semester*)** |  |  |
| Freshman | 370 | 82.2 |
| Transfer Student | 75 | 16.7 |
| **Living Arrangements (Campus)** |  |  |
| On Campus | 341 | 29.6 |
| Off Campus (walking distance) | 279 | 24.2 |
| Off Campus (drive-public transit) | 513 | 44.5 |
| Other | 19 | 1.6 |
| **College Major^*^** |  |  |
| Communication | 307 | 26.6 |
| Business/Pre-Business | 131 | 11.3 |
| Computer Science | 72 | 6.3 |
| Psychology | 52 | 4.5 |
| Nursing/Pre-nursing | 38 | 3.2 |
| Marketing/Advertising | 35 | 3.1 |
| Hospitality | 29 | 2.5 |
| Criminal Justice | 26 | 2.3 |
| Accounting | 24 | 2.1 |
| Finance | 22 | 1.9 |
| Biology/Biological Sciences | 20 | 1.7 |
| Kinesiology | 19 | 1.6 |
| Undecided | 15 | 1.3 |
| **Notes.** *N* = 1152. *^*^*Students were asked if they were a communication major (yes/no) and if no, their major. Only majors that reached at least 1% reported above. | | |
